# Supplementary material for: Identifying the Brain Circuits that Regulate Pain-Induced Sleep Disturbances
Source: bioRxiv. 2024 Dec 20:2024.12.20.629596. Preprint. [Version 1] doi: 10.1101/2024.12.20.629596 (PMC11702673; doi:10.1101/2024.12.20.629596)
Supplement: Supplement 1 [file NIHPP2024.12.20.629596v1-supplement-1.pdf]

bioRxiv preprint doi: https://doi.org/10.1101/2024.12.20.629596; this version posted December 20, 2024. The copyright holder for this preprint (which was not certified by peer review) is the author/funder, who has granted bioRxiv a license to display the preprint in perpetuity. It is made available under aCC-BY 4.0 International license.

Supplementary Figures

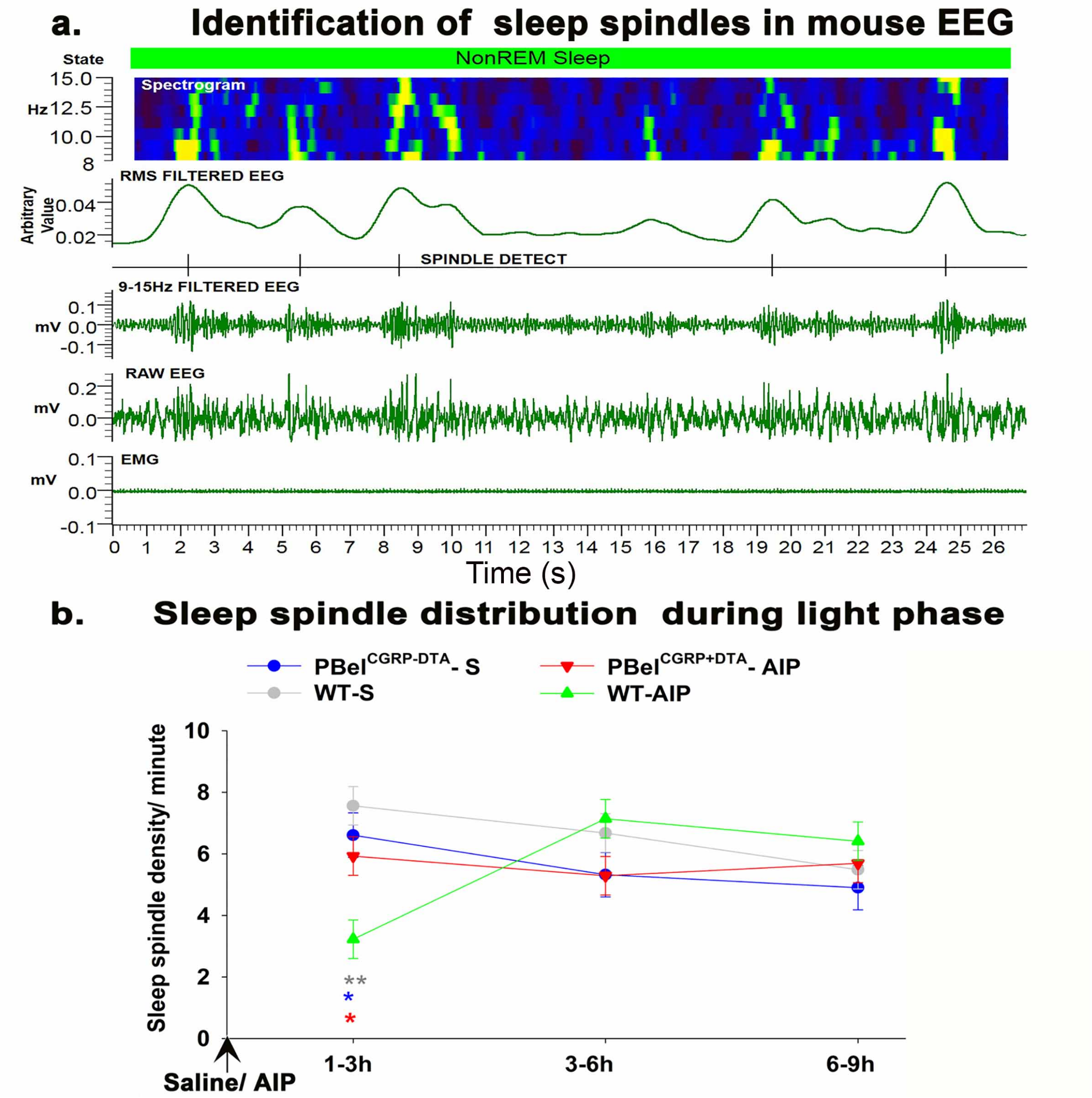

Figure S1.

bioRxiv preprint doi: https://doi.org/10.1101/2024.12.20.629596; this version posted December 20, 2024. The copyright holder for this preprint (which was not certified by peer review) is the author/funder, who has granted bioRxiv a license to display the preprint in perpetuity. It is made available under aCC-BY 4.0 International license.

Figure legends for the supplementary figures:

Supplementary Figure 1 (Fig. S1). *Depiction of sleep spindles in a representative EEG recording and distribution of sleep spindles across light phase.* A representative photomicrograph of the EEG and EMG recording from a mouse showing the filtered EEG and spectrogram used to identify sleep spindles (**a**). Graph showing the sleep-spindle density (spindles/min) in 3h bins after saline injection or AIP in WT and PBel$^{CGRP\text{-}DTA}$ mice (**b**). The groups were compared using a two-way (treatment X time) ANOVA, followed by Holms-sidak method for multiple comparisons, where **- P<0.001; *- P<0.05. The color of the asterisk represents the comparison group.

bioRxiv preprint doi: https://doi.org/10.1101/2024.12.20.629596; this version posted December 20, 2024. The copyright holder for this preprint (which was not certified by peer review) is the author/funder, who has granted bioRxiv a license to display the preprint in perpetuity. It is made available under aCC-BY 4.0 International license.

Validation of CGRP-ChR2 mice with expression of ChR2-mcherry in the CGRP neurons, fibers and terminals

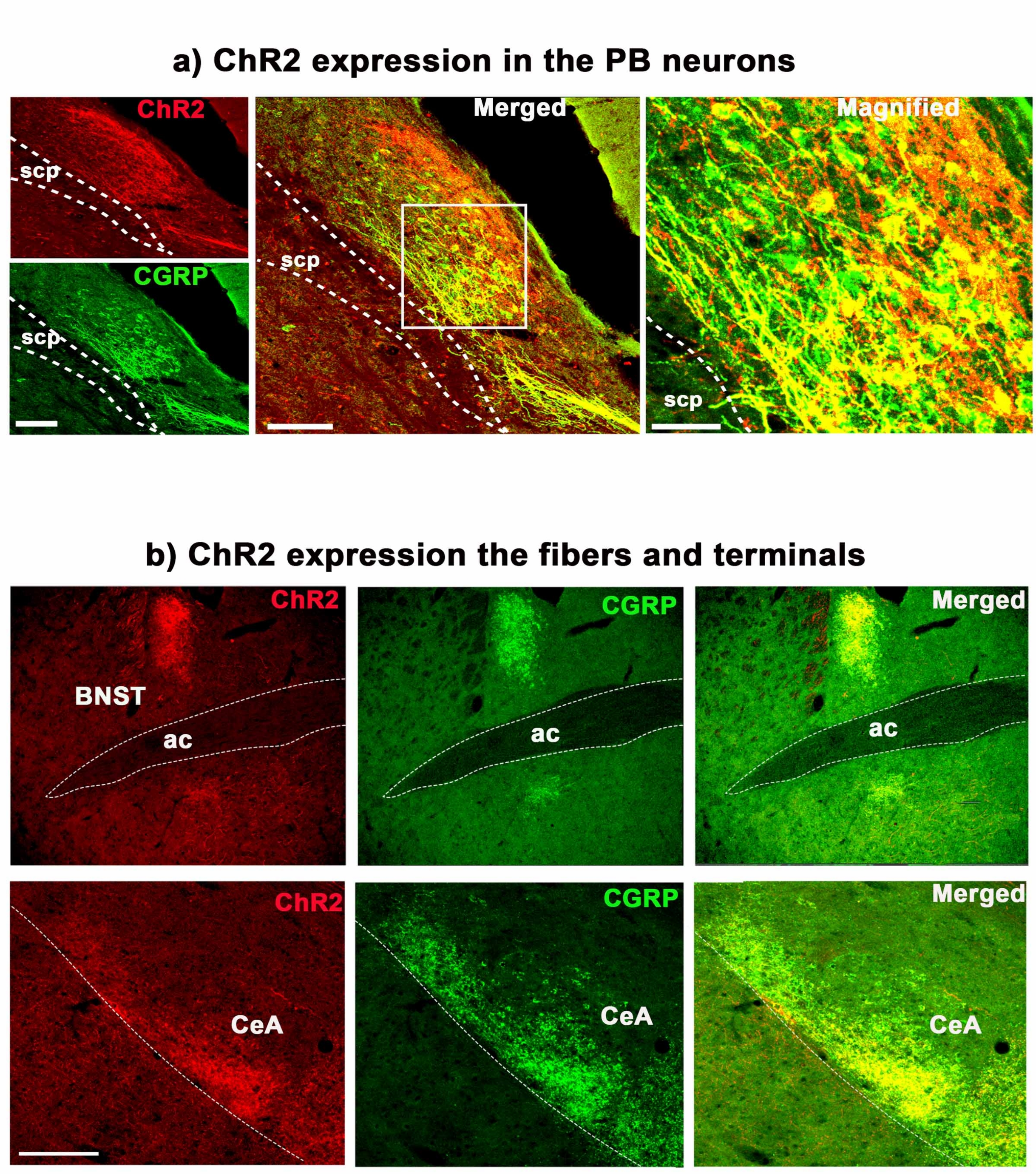

Figure S2

bioRxiv preprint doi: https://doi.org/10.1101/2024.12.20.629596; this version posted December 20, 2024. The copyright holder for this preprint (which was not certified by peer review) is the author/funder, who has granted bioRxiv a license to display the preprint in perpetuity. It is made available under aCC-BY 4.0 International license.

Supplementary Figure 2 (Fig. S2): *Validation of Channel Rhodopsin (ChR2) expression in the CGRP expressing neurons and terminal fields in the CGRP-ChR2 mice:* Photo-micrographs showing brain sections from the CGRP-ChR2 mice, immuno-stained for mcherry to label ChR2 (red) and CGRP (green) in the PBel neurons (**a**) and also in the CGRP expressing fibers and terminals seen in BNST and CeA (**b**). Scale: 100μm in a (left) and b; 60 μm and 30 μm in middle and right panels in a.
